# Prevalence and co‐occurrence of dementia risk factors in Denmark: a nationwide study

**DOI:** 10.1002/alz70860_100738

**Published:** 2025-12-23

**Authors:** Janet Janbek, Thomas Munk Laursen, Kasper Jørgensen, Martin Mejlby Jensen, Marie Holm Eliasen, Anne Illemann Christensen, Sebastian Walsh, Andrew Sommerlad, Carol Brayne, Gunhild Waldemar

**Affiliations:** ^1^ Danish Dementia Research Centre, Copenhagen University Hospital ‐ Rigshospitalet, Copenhagen, Denmark; ^2^ National Centre for Register‐Based Research, Aarhus University, Aarhus, Aarhus, Denmark; ^3^ Danish Dementia Research Centre, Copenhagen Ø, 2100, Denmark; ^4^ DEFACTUM, Central Denmark Region, Aarhus, Aarhus, Denmark; ^5^ Center for Clinical Research and Prevention, Bispebjerg and Frederiksberg Hospital, Copenhagen, Copenhagen, Denmark; ^6^ National Institute of Public Health, University of Southern Denmark, Copenhagen, Copenhagen, Denmark; ^7^ Cambridge Public Health, University of Cambridge, Cambridge, Cambridgeshire, United Kingdom; ^8^ University College London, London, London, United Kingdom; ^9^ Department of Clinical Medicine, University of Copenhagen, Copenhagen, Denmark; ^10^ Danish Dementia Research Centre, Dept. of Neurology, Copenhagen University Hospital ‐ Rigshospitalet, Copenhagen, Denmark

## Abstract

**Background:**

Modifiable dementia risk factors have rarely been comprehensively investigated in a national population and have mostly been studied for their individual impact on dementia risk. However, the high prevalence of multimorbidity, clustering and co‐occurrence of several risk factors is common, and the interplay may affect dementia risk with implications for policies targeting risk reduction. Population‐based and multidomain interventions may be desirable but currently rely on limited evidence of the co‐occurrence of dementia risk factors at the population level. Thus, we aimed to explore the prevalence of sixteen modifiable risk factors for dementia, focusing on the most frequent combinations of these risk factors.

**Method:**

Information on risk factors was drawn from nationwide registries and the 2010/2013 Danish National Health Survey (DNS). The main population was a closed cohort of individuals ≥65 years on 1 January 2022. A subpopulation comprising individuals with information on risk factors unavailable in the registries was drawn from DNS. Period prevalence was calculated for the risk factors and all possible risk factor combinations occurring in ≥10% of the subpopulation.

**Result:**

The main population comprised 1,214,286 individuals, and 88,565 had DNS data (majority were women; the median birth cohort was 1948). Among the 88,565 individuals, 4828 (5%) had no risk factors, 10,929 (12%) had only one risk factor, and 72,899 (82%) had more than one. Hypertension was the most prevalent (57%), followed by a history of infections requiring hospitalization (52%), hypercholesterolemia (50%), and cardiovascular disease (46%), and these formed the most common combinations. The least prevalent was vision impairment (2%). Men had a higher prevalence of all risk factors except for having lower depression and more education. All risk factors were more commonly first recorded at age <65 except CVD, hearing loss, diabetes, sleep disorders, and vision impairment which were more commonly first recorded after age ≥65.

**Conclusion:**

Cardiometabolic risk factors were the most prevalent, as were their combinations. Clustering of risk factors is common, with 82% of the population having multiple risk factors. Findings emphasize the need to focus on population‐level interventions for dementia risk reduction that account for the clustering of risk.

